# Targeting Rb Mutant Cancers by Inactivating TSC2

**DOI:** 10.18632/oncotarget.130

**Published:** 2010-07-26

**Authors:** Jennifer S. Searle, Binghui Li, Wei Du

**Affiliations:** Ben May Department for Cancer research 929 E. 57th St, Chicago, IL 60637, USA

**Keywords:** Rb, TSC2, ROS, TOR, SOD2, synthetic lethal

## Abstract

Retinoblastoma (Rb), a tumor suppressor gene, is inactivated in many types of cancer. However little is known about how the loss of Rb function can be targeted in cancer therapies. We have identified that inactivation of TSC2 in Rb mutant cancer cells will induce a synergistic cell death. The synergistic cell death is due to an increase in cellular stress including, metabolic, ER, and oxidative stress. Therefore, inactivation of TSC2 and chemothereputics that result in induction of cellular stress may be a novel and effective way to treat cancers containing inactivated Rb.

## INTRODUCTION

Conventional cancer treatments, such as inhibitors of S phase or mitosis, target all the proliferating cells and therefore cause many side effects. The new “targeted” chemotherapy drugs, such as imatinib, which attack tumor cells by one specific mechanism, are generally safer and have fewer side effects; but, have typically shown initial success followed by recurrence and resistance. Successful treatment of cancers generally requires the use of multiple chemotherapeutic agents, but physicians are limited by the limited number of mechanistically unique chemotherapy drugs available. Therefore, identification of new drugs that target distinct properties of cancers is critically needed.

Defects in cancer cells that lead to uncontrolled proliferation usually include both deregulated oncogenic activation as well as inactivated tumor suppressors. The deregulated oncogenic signaling components are well recognized to be potential targets for new cancer drugs since blocking the deregulated oncogenic signaling inhibits cell proliferation and induces cell death. In contrast, loss of tumor suppressors in cancers is not easily exploited for developing cancer therapeutics due to the lack of approaches to restore the lost tumor suppressor function in all cancer cells. An alternative approach to restoring the tumor suppressor function in cancer cells is to specifically kill cancer cells that have the inactivated tumor suppressor. However, it is difficult to identify genes that will cause synthetic lethality (induction of cell death only when both the tumor suppressor and the gene are inactivated). Our lab has developed a screen to identify genes that are synthetic lethal in combination with the tumor suppressor gene Retinoblastoma (Rb), using the model system *Drosophila melonogaster* [1].

## INACTIVATION OF TSC2 AND RB LEADS TO SYNERGISTIC CELL DEATH INDUCTION

**Fig. 1:**
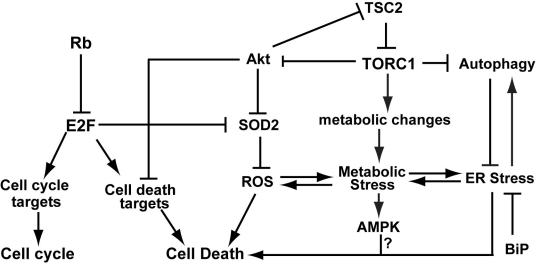
A model of inactivation of Rb and TSC2 results in synergistic cell death. Inactivation of TSC2 results in deregulation of TORC1 signaling, which in turn leads to increased cellular stress (including metabolic stress, ROS, and ER stress) and decreased Akt survival signaling. Inactivation of Rb leads to activation of E2F transcription factors which in turn results in the upregulation of genes that promote cell death as well as a failure to upregulate genes (such as SOD2) that protect cells from increased cellular stress. Our model suggests that inactivation of Rb and TSC2 leads to an accumulation of cellular stress as well as cell death signals, resulting in synergistic cell death induction.

Retinoblastoma or Rb is a tumor suppressor protein that was first identified in children that exhibited tumors of the retina. Rb functions primarily by binding to the E2F transcription factors, which are regulated by the cyclin dependent kinases. E2F proteins regulate the transcription of many types of genes including genes involved in cell cycle, DNA repair, differentiation, and metabolism (reviewed in [2, 3]). The Rb-E2F pathway also plays a role in apoptosis by regulating the transcription of cell death targets such that Rb mutant cells are sensitized for cell death (Fig.1) [4]. Since Rb and other components of the Rb signaling pathway are often found to be inactivated in a variety of cancers, loss of Rb is a good candidate for targeted drug therapies.

Using the screen in *Drosophila* to identify Rb synthetic lethal genes, we reported the identification of a mutation in the TSC2 homologue (gigas) that caused a synergistic cell death induction with loss of the Rb homogogue in *Drosophila* [1].

Mutations in TSC2 (or TSC1) result in a disease called Tuberous sclerosis that causes benign tumors in several different organs as well as neural defects (reviewed in [5-7]). TSC1 and TSC2 are part of the TOR signaling pathway which is responsible for sensing nutrient availability and growth factors and inducing the appropriate metabolic changes for the cell. The TSC complex is a GAP (GTPase activating protein), which promotes the conversion of GTP-bound Rheb to the inactive GDP-bound Rheb. Inactivation of TSC2 leads to deregulated TORC1 activity, which promotes protein synthesis, induces increased oxidative phosphorylation and cellular stress, and inhibits autophagy (Fig. 1) [6].

To test whether inactivation of TSC2 can be used to specifically kill Rb mutant cancers, we used lentivirus to express short hairpin RNA specific to TSC2 (shTSC2) in Rb mutant and Rb wild type cancer cells. Our results showed that shTSC2 increased cell death under a variety of stress conditions and inhibited the growth of Rb mutant cells in soft agar and in mouse xenografts [1]. These results suggested that TSC2 is a good protein to inactivate in order to specifically kill Rb mutant cancers.

## MECHANISMS OF CELL DEATH IN TSC2 INACTIVATED-RB MUTANT CANCER CELLS

Inactivation of TSC2 resulted in a decreased level of Akt signaling. PI3K/Akt is a major component of survival signaling that modulates E2F-mediated cell death [8], suggesting that decreased PI3K/Akt survival signaling may contribute to shTSC2-induced cell death. In support of this idea, microarray analysis revealed that shTSC2 significantly reduced expression of components of the insulin-like growth factor/EGF/PI3K survival signaling pathways and reduced the level of survival factors such as Bcl-XL [1]. Additionally, expression of Bcl-XL significantly decreased shTSC2 induced cell death; suggesting that the reduced survival signaling contributes to shTSC2- induces cell death in Rb mutant.

However, decreasing the level of survival signaling is not the only and perhaps not the primary mechanism by which shTSC2 induces cell death as overexpression of activated Akt failed to inhibit cell death in Rb, TSC2 inactivated cells [1]. While Akt can protect cells from undergoing cell death induced by a variety of cell death signals, AKT does not protect cells from cell death induced by ROS [9]. Indeed inactivation of both TSC2 and Rb synergistically induce a high level of cellular stress, including oxidative stress and ER stress, which are critical for synergistic cell death induction. In both flies and human cancer cells, synergistic cell death is E2F-dependent and mediated by enhanced protein synthesis due to increased TORC1 activity. Decreasing the level of protein synthesis, either by inactivation of S6K in flies or by treatment of cancer cells with G418, significantly inhibited synergistic cell death induction [1]. These results suggest that enhanced protein synthesis is a key contributor of shTSC2-induced cell death.

**Fig. 2:**
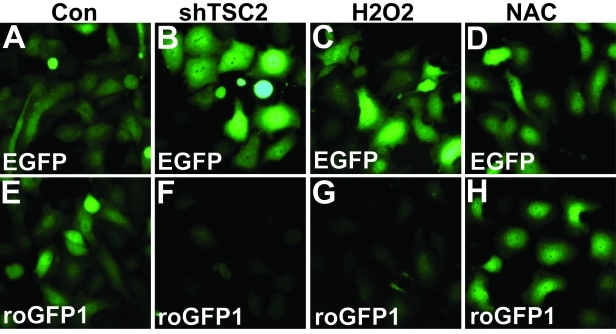
TSC2 knockdown in Rb mutant cancer cells induces an increase in ROS. roGFP1 is a redox-sensitive variant of EGFP which exhibits a decrease in florescence intensity in an oxidative state. Du145 cells containing either EGFP or roGFP1 were treated as shown. A-D) The fluorescence intensity of EGFP did not vary with redox status. E-H) The intensity of the florescent signal of roGFP decreased when cells were in an oxidative state (H2O2, G) as compared to control (E) but remained the same when treated with NAC, an inhibitor of ROS (H). Knocking down TSC2 (shTSC2) resulted in an oxidative state in the cells (compare E and F). For experimental details, see reference [1].

Enhanced protein synthesis can lead to low ATP levels and increased metabolic stress, which in turn can induce increased oxidative phosphorylation in the mitochondria and decreased lipid synthesis. Increased oxidative phosphorylation causes an increased level of reactive oxygen species (ROS). To show that shTSC2 induces an oxidative state to the Rb mutant cancer cells, (Du145), we used a redox sensitive form of EGFP (roGFP), which loses florescence in the oxidative state. As shown in Fig. 2, while neither H2O2 nor antioxidant NAC significantly affect EGFP fluorescence signal (Fig. 2A-D), H2O2 significantly decreased fluorescence signal from roGFP1 (Fig. 2G). Interestingly, shTSC2 significantly decreased the roGFP1 but not the EGFP signal (Fig. 2B and F), supporting the idea that inactivation of TSC2 induces an oxidative state in the cells.

Cancer cells often exhibit increased *de novo* lipid synthesis. In fact, inhibition of *de novo* lipid or cholesterol synthesis can suppress tumor cell growth and induce cell death [10-12]. Interestingly, microarray analysis revealed that shTSC2 led to significant inhibition of the key enzymes in lipid synthesis, but not in the adapted cells that developed resistance to shTSC2-induced cell death. Furthermore, an increased level of ER stress is observed in TSC2 knockdown Rb mutant cells but not in the adapted cells [1]. These results suggested that an inhibition of lipid synthesis in conjunction with increased protein synthesis induces an increased level of ER stress, which may also contribute to synergistic cell death.

While TSC2 inactivation plays a key role in inducing cellular stress, it appears that Rb plays an important role in the cells ability to deal with increased cellular stress. SOD2, AMPKα2, and GRP78/BiP, which are important regulators of oxidative stress, metabolic stress, and ER stress, respectively; have been reported to be targets of the E2F transcription factors [8, 13, 14]. We showed that Rb regulates SOD2 expression. In the absence of Rb, SOD2 expression failed to be upregulated by shTSC2 [1]. Therefore, Rb mutant cells accumulate high levels of ROS upon TSC2 knockdown and are more sensitive to shTSC2- induced cell death. It will be interesting to test if AMPKα2 and GRP78/BiP are also regulated by Rb and contribute to Rb and TSC2 inactivation induced cell death.

In flies we found that inhibiting the JNK signaling pathway, which is the key stress signaling pathway in flies, led to a decrease in cell death in the cells mutant for the homologs of Rb and TSC2 [1]. These results suggested that in both flies and mammalian cells the inhibition of TSC2 function can lead to increased stress signaling that in turn leads to an increase in cell death.

In summary, we propose a model in which Rb mutant cells are (1) already sensitized to apoptosis as inactivation of Rb can lead to upregulation of E2F mediated transcription of apoptotic factors and (2) sensitized to increased cellular stress due to defects in the regulation of proteins that modulate cellular stress such as SOD2 (Fig. 1). Therefore, inactivation of TSC2 which results in increased cellular stress including oxidative, ER and metabolic stress can “tip the scale” toward induction of cell death.

## THERAPIES FOR RB MUTANT CANCERS

Our model clearly illustrates the interconnectedness of metabolic regulation, cell stress, proliferation and apoptotic signaling pathways. Cancer cells are known to have altered metabolism, exhibiting enhanced aerobic glycolysis and *de novo* lipid synthesis [3, 12, 15]. Interestingly, some of the same proteins that regulate cell proliferation such as oncogenes, tumor suppressors and cell cycle checkpoint proteins also regulate metabolic processes in the cell [3, 16, 17]. The altered metabolism in cancer cells potentially offers new drug targets for the treatment of cancer and these types of new drug targets will likely be useful for simultaneously targeting multiple pathways to efficiently induce cell death and to prevent the development of resistance. We observed that inactivation of Rb and TSC2 synergistically induce cell death due to cellular stress induced by altered metabolism, suggesting that agents that target the metabolic pathways may enhance TSC2 inactivation induced specific killing of the Rb mutant cancer cells by inducing increased cellular stress.

Rb is inactivated in a large number of cancers, making Rb a potentially ideal candidate for finding treatments for these cancers. Since shRNA will be difficult to use in cancer treatment, it is likely that small molecule inhibitors of TSC2 function will need to be developed for the treatment of Rb mutant cancers.

In previous clinical trials it was found that inhibiting kinases in cancer treatment often leads to the development of resistant cancers. It is possible that an inhibitor of TSC2 can be used in conjunction with drugs that target deregulated oncogenic activation to decrease the development of resistant cancers and achieve a better clinical outcome in Rb mutant cancers. We showed that inactivation of Rb and TSC2 induced cell death was mediated by increased cellular stress. Therefore, we suggest that agents that can modulate cellular stress, such as an activator of AMPK or inhibitors of lipid synthesis, could potentially enhance the killing of Rb mutant cancer cells by inhibiting TSC2. In addition, since apoptosis induced by shTSC2 in Rb mutant cells is affected by Bcl-Xl levels, inhibitor of Bcl-2 family of proteins, such as ABT-263 or ABT-737 [18], [19], could also increase the efficacy of TSC2 inhibitor induced killing of Rb mutant cancers.
